# Supplementary material for: End Sequence Analysis Toolkit (ESAT) expands the extractable information from single-cell RNA-seq data
Source: Genome Res. 2016 Oct;26(10):1397–410. doi: 10.1101/gr.207902.116 (PMC5052061; doi:10.1101/gr.207902.116)
Supplement: Supplemental Material [file supp_gr.207902.116_Supp_Materials.tar.gz › ESAT-esat_v0.1/doc/html/classbroad_1_1pda_1_1geneexpression_1_1clustering_1_1_kmeans.html]

ESAT: broad.pda.geneexpression.clustering.Kmeans Class Reference


ESAT


- Main Page
- Related Pages
- Packages
- Classes
- Files

- Class List
- Class Index
- Class Hierarchy
- Class Members

All Classes Namespaces Files Functions Variables Enumerator Pages

- broad
- pda
- geneexpression
- clustering
- Kmeans

Classes |
Public Member Functions |
Static Public Member Functions |
List of all members

broad.pda.geneexpression.clustering.Kmeans Class Reference

Collaboration diagram for broad.pda.geneexpression.clustering.Kmeans:

[legend]

|  |  |
| --- | --- |
| Classes | |
| class | **KmeansInfo** |
|  | |
| class | **SilhouetteInfo** |
|  | |

|  |  |
| --- | --- |
| Public Member Functions | |
|  | Kmeans (MatrixWithHeaders mat, MatrixWithHeaders initCentroids, int numOfClusters, String distanceMetric) |
|  | |
| void | runKmeans () |
|  | |
| void | calcSilhouette () |
|  | |
| void | calcLightSilhouette () |
|  | |
| double | getSilhouette () |
|  | |
| void | writeClusters (BufferedWriter bw) throws IOException |
|  | |
| void | writeClustersCentroids (BufferedWriter bw) throws IOException |
|  | |
| void | writeDistanceFromCentroids (BufferedWriter bw) throws IOException |
|  | |
| void | writeDistanceFromInputCentroids (BufferedWriter bw) throws IOException |
|  | |
| KmeansInfo | getClusterInfo () |
|  | |
| void | writeDistanceFromInputCentroids (MatrixWithHeaders centroids, BufferedWriter bw) throws IOException |
|  | |
| void | writeSilhouetteRes (int currK, BufferedWriter bwGeneS, BufferedWriter bwAllS, BufferedWriter bwClusterS) throws IOException |
|  | |

|  |  |
| --- | --- |
| Static Public Member Functions | |
| static double | distance (double[] a, double[] b, String metric) |
|  | |

## Constructor & Destructor Documentation

|  |  |  |  |
| --- | --- | --- | --- |
| broad.pda.geneexpression.clustering.Kmeans.Kmeans | ( | MatrixWithHeaders | *mat*, |
|  |  | MatrixWithHeaders | *initCentroids*, |
|  |  | int | *numOfClusters*, |
|  |  | String | *distanceMetric* |
|  | ) |  |  |

Here is the call graph for this function:

## Member Function Documentation

|  |  |  |  |  |
| --- | --- | --- | --- | --- |
| void broad.pda.geneexpression.clustering.Kmeans.calcLightSilhouette | ( |  | ) |  |

|  |  |  |  |  |
| --- | --- | --- | --- | --- |
| void broad.pda.geneexpression.clustering.Kmeans.calcSilhouette | ( |  | ) |  |

|  |  |  |  |
| --- | --- | --- | --- |
| static double broad.pda.geneexpression.clustering.Kmeans.distance | ( | double[] | *a*, |
|  |  | double[] | *b*, |
|  |  | String | *metric* |
|  | ) |  |  |

static

Here is the call graph for this function:

|  |  |  |  |  |
| --- | --- | --- | --- | --- |
| KmeansInfo broad.pda.geneexpression.clustering.Kmeans.getClusterInfo | ( |  | ) |  |

|  |  |  |  |  |
| --- | --- | --- | --- | --- |
| double broad.pda.geneexpression.clustering.Kmeans.getSilhouette | ( |  | ) |  |

|  |  |  |  |  |
| --- | --- | --- | --- | --- |
| void broad.pda.geneexpression.clustering.Kmeans.runKmeans | ( |  | ) |  |

Here is the call graph for this function:

|  |  |  |  |  |  |
| --- | --- | --- | --- | --- | --- |
| void broad.pda.geneexpression.clustering.Kmeans.writeClusters | ( | BufferedWriter | *bw* | ) | throws IOException |

|  |  |  |  |  |  |
| --- | --- | --- | --- | --- | --- |
| void broad.pda.geneexpression.clustering.Kmeans.writeClustersCentroids | ( | BufferedWriter | *bw* | ) | throws IOException |

|  |  |  |  |  |  |
| --- | --- | --- | --- | --- | --- |
| void broad.pda.geneexpression.clustering.Kmeans.writeDistanceFromCentroids | ( | BufferedWriter | *bw* | ) | throws IOException |

|  |  |  |  |  |  |
| --- | --- | --- | --- | --- | --- |
| void broad.pda.geneexpression.clustering.Kmeans.writeDistanceFromInputCentroids | ( | BufferedWriter | *bw* | ) | throws IOException |

|  |  |  |  |
| --- | --- | --- | --- |
| void broad.pda.geneexpression.clustering.Kmeans.writeDistanceFromInputCentroids | ( | MatrixWithHeaders | *centroids*, |
|  |  | BufferedWriter | *bw* |
|  | ) |  | throws IOException |

Here is the call graph for this function:

|  |  |  |  |
| --- | --- | --- | --- |
| void broad.pda.geneexpression.clustering.Kmeans.writeSilhouetteRes | ( | int | *currK*, |
|  |  | BufferedWriter | *bwGeneS*, |
|  |  | BufferedWriter | *bwAllS*, |
|  |  | BufferedWriter | *bwClusterS* |
|  | ) |  | throws IOException |

Here is the call graph for this function:

---

The documentation for this class was generated from the following file:

- java/broad/pda/geneexpression/clustering/Kmeans.java


---

Generated on Fri Aug 15 2014 15:11:01 for ESAT by  

 1.8.7
